# Randomised Clinical Trial: Calorie Restriction Regimen with Tomato Juice Supplementation Ameliorates Oxidative Stress and Preserves a Proper Immune Surveillance Modulating Mitochondrial Bioenergetics of T-Lymphocytes in Obese Children Affected by Non-Alcoholic Fatty Liver Disease (NAFLD)

**DOI:** 10.3390/jcm9010141

**Published:** 2020-01-04

**Authors:** Rossella Negri, Giovanna Trinchese, Fortunata Carbone, Maria Grazia Caprio, Giovanna Stanzione, Carmen di Scala, Teresa Micillo, Francesco Perna, Luca Tarotto, Monica Gelzo, Gina Cavaliere, Maria Immacolata Spagnuolo, Gaetano Corso, Giuseppina Mattace Raso, Giuseppe Matarese, Maria Pina Mollica, Luigi Greco, Raffaele Iorio

**Affiliations:** 1 European Laboratory for the Study of Food Induced Diseases (ELFID), 80131 Naples, Italy; rosnegri@unina.it (R.N.); mispagnu@unina.it (M.I.S.); ydongre@unina.it (L.G.); riorio@unina.it (R.I.); 2 Department of Translational Medical Sciences, Section of Paediatrics, University of Naples Federico II, 80131 Naples, Italy; gio.stanzione@hotmail.it (G.S.); carmendiscala@gmail.com (C.d.S.); 3 Department of Biology, University of Naples Federico II, 80126 Naples, Italy; giovanna.trinchese@unina.it (G.T.); teresa.micillo2@unina.it (T.M.); gina.cavaliere@unina.it (G.C.); 4 Institute for Experimental Endocrinology and Oncology, National Research Council (IEOS-CNR), 80131 Naples, Italy; f.carbone@ieos.cnr.it (F.C.); giuseppe.matarese@unina.it (G.M.); 5 Neuroimmunology Unit, IRCCS Fondazione Santa Lucia, 00143 Rome, Italy; 6 Institute of Biostructure and Bioimaging IBB, CNR, 80128 Naples, Italy; mariagrazia.caprio@ibb.cnr.it; 7 Department of Clinical Medicine and Surgery, University of Naples Federico II, 80131 Naples, Italy; francesco.perna@unina.it; 8 Department of Advanced Biomedical Sciences, University of Naples Federico II, 80131 Naples, Italy; luca.tarotto@gmail.com; 9 Department of Clinical and Experimental Medicine, University of Foggia, 71122 Foggia, Italy; gaetano.corso@unifg.it; 10 CEINGE- Biotecnologie Avanzate S.c.a r.l., 80145 Naples, Italy; monica.gelzo@unina.it; 11 Department of Pharmacy, University of Naples Federico II, 80131 Naples, Italy; mattace@unina.it; 12 Department of Molecular Medicine and Medical Biotechnology, University of Naples Federico II, 80131 Naples, Italy

**Keywords:** NAFLD, pediatric hepatology, inflammation, immunology, nutrition

## Abstract

Fatty liver disease is a serious complication of childhood obesity. Calorie-restricted regimen (RCR) is one of the effective therapy for this condition. Aim of the study was to evaluate the effect of lycopene-rich tomato sauce with oregano and basil extracts in obese children with fatty liver on RCR. 61 obese children with fatty liver were enrolled, 52 completed the study. A randomized cross over clinical trial was performed. Participants were assigned to RCR alone or with a supplement of lycopene-rich tomato juice for 60 days; subsequently, the groups were switched to the alternative regimen for the next 60 days. Reduction in BMI, HOMA-IR, cholesterol, triglycerides, liver size, and steatosis was more profound in tomato-supplemented group. Leptin decreased in both groups whereas adiponectin raised only after tomato supplementation. RCR is associated with the impaired engagement of T-cells glycolysis and proliferation, tomato-supplementation resulted in glycolytic metabolic activation of T-cells. Tomato juice ameliorates glucose and lipid metabolism in obese children, improve oxidative and inflammatory state and modulates the mitochondrial metabolism of T-cells contributing to a maintenance of a proper immune surveillance in children, impaired by RCR. The addition of tomato to RCR could be considered a protective and preventive support to obese child.

## 1. Introduction

Non-alcoholic fatty liver disease (NAFLD) is the most common cause of liver dysfunction in obese children. Liver infiltration with fats progressively leads to hepatocyte injury, due to oxidative stress, mitochondrial dysfunction and inflammation [1,2].

Increased LDL and decreased HDL serum levels correlate with intrahepatic lipid content generating fatty liver-associated dyslipidaemia, a well-known risk factor for early cardiovascular diseases [3]. In NAFLD, aminotransferase activity marks the progression of liver damage, together with inflammatory biomarkers [4].

In children with hepatic steatosis, lifestyle interventions, including calorie restriction, physical activity, and exercise, are actually considered the primary therapy for the management of NAFLD [5]. Inconclusive and conflicting results ensue from studies on pharmacological intervention [6].

Africa et al. [7] recently reviewed twenty-eight studies of lifestyle interventions on children with NAFLD. Among them, only a minority achieved a significant improvement of hepatic outcome measures, such as serum alanine aminotransferase (ALT), and a hepatic fat fraction or histological scores. The authors concluded that due to the heterogeneity of intervention strategies and outcome measures as well as controversial results, no specific intervention turned out to be of particular benefit for the majority of patients.

Less well understood, however, are the effects of systemic metabolism or whole organism nutritional status on immune cell function and metabolism. Indeed, the activation of different metabolic pathways in immune cells are able to control the induction of functionally distinct T cell subsets toward a pro- or anti-inflammatory phenotype [8,9]. In addition, conditions such as obesity is associated with increased expression of mediators such as IL-6 and leptin that promote inflammation and may worsen NAFLD [10]. Several studies suggest that RCR might compromise the host’s defense against pathogenic infection and result in higher morbidity and mortality. However RCR associated to Mediterranean style adoption could represent a proper compromise.

Notably, adherence to the Mediterranean diet reduces the risk of non-alcoholic steatohepatitis (NASH) and diabetes in pediatric patients with obesity: indeed a study on 243 Italian obese children indicates a correlation between poor adherence to the Mediterranean diet and liver damage and glucose imbalance [11].

Tomato, a cornerstone food of the Mediterranean diet, is an optimal source of bioactive compounds such as carotenoids, polyphenols and vitamin E, which are resistant to ordinary storing and cooking procedures, and vitamin C, that is partially lost during processing. In a rat model of NAFLD, the addition of tomato juice significantly improved the metabolic profile altered by the high fat diet [12].

Lycopene, the main carotenoid component of tomato, has a strong antioxidant effect in vivo, protecting biomolecules from the oxidative stress [13].

After absorption, lycopene is preferentially stored in the liver, where it can exert its function [14]. Here, lycopene reduces the development of hepatic steatosis induced by a high-fat diet (HFD) in experimental NASH models [15]. The key role of this carotenoid against fatty liver was confirmed by the reduced plasma lycopene levels in subjects affected by NASH, suggesting a possible link between low lycopene levels and the development of liver diseases [16]. Consistently, in humans, tomato juice consumption improves blood antioxidant defences and decreases inflammatory biomarkers linked to obesity and metabolic syndrome [17].

Indeed, tomatoes are generally consumed in Mediterranean diet in combination with basil and oregano, which have strong antioxidant activity and a high concentration of phenolic compounds. Preclinical studies have shown that basil extracts decreased the levels of serum Thiobarbituric Acid Reactive Substances (TBARS), AST and ALT, restored hepatic antioxidant levels and reduced the levels of lipid peroxidation in rats [18].

The first objective of this study is to evaluate the effect of a lycopene-enriched tomato juice on liver damage in obese children affected by NAFLD. A further aim of this study is to investigate the influence of systemic nutritional status on T cell proliferation and function.

## 2. Methods

### 2.1. Participants and Study Design

Clinical Trial Registry number and website NCT03463967 https://clinicaltrials.gov. The parents of the participating children gave their informed consent for inclusion before they participated in the study. The study protocol conforms to the ethical guidelines of the 1975 Declaration of Helsinki as reflected in a priori approval by the Ethical Committee of University Federico II of Naples N.170/16 of the 09.05.2016. 

Obese children with NAFLD referred to the Liver Unit of the Department of Clinical Pediatric of the University Federico II of Naples were eligible, on the basis of:

- Age 4–14 years
- BMI > 85th percentile
- Liver steatosis evaluated as mild, moderate or severe by US (hyperechogenic liver tissue compared with the adjacent kidney cortex)

Patients were excluded in case of:

- Other liver disease, such as viral hepatitis, autoimmune hepatitis, Wilson’s disease, alpha-1-antitrypsin deficiency
- Diabetes or manifest metabolic alterations
- Other associated disease

To provide 80% power to detect 25% or greater relative shift of outcome variables, with a first-degree error of 05, a sample of 50 cases was estimated for a crossover trial. This is a randomized, crossover, one side open trial with blinded outcome evaluation. Participants were obviously aware of the treatment added to the diet. The pediatric team, the care providers, the US specialist, the LAB technicians were all blind to the treatment. A statistician, blind to the treatment, generated the randomized sequence to allocate the first treatment (diet + tomato or diet only) and performed the analyses. The randomization was obtained by odds/even numbers: a closed envelope containing the allocation for each patient.

Patients were recruited from October 2016 to January 2018; the follow-up (2 months) took place from March 2017 to July 2018. They were followed by a pediatric team at the outpatient department of the Department of Pediatrics of the University of Naples Federico II. Informed consent was obtained from the parents of the participating children.

At enrollment, all participants underwent a washout low carotenoids diet for two weeks (T0), then they were randomly assigned to a restricted calorie regimen (RCR) alone (group 1 = 27) or with tomato juice supplementation together with basil and oregano extract in extra virgin olive oil (RCR + Tomato) (group 2 = 34) for 60 days (T1); subsequently, in the crossover phase, they were switched to the alternative regimen (RCR: 26 or RCR + Tomato: 26) for the next 60 days (T2). At the end of this period, all the subjects were kept on RCR for 60 days more (T3). (Figure S1—Consort Flow Diagram; Supplementary material—Randomized Crossover Trial Protocol). 

About 1200 kcal were daily administered to all children in RCR by a Mediterranean style diet. When supplemented with tomato juice, children received 100 mL of lycopene-enriched (0.011%) tomato juice daily (‘Lycolife’, La Fiammante, Buccino, Italy), containing 1 mL of vitamin D-enriched (227 µg) extra virgin olive oil with eugenol, carvacrol, and thymol (10 μg/mL) essential components of oregano and basil. A dedicated dietitian checked the compliance to the diet once a week, irrespective of the treatment, for the whole study.

At T1, T2, and T3, a 3-days dietary recall was performed by direct interview with children and guardians. The WinFood Pro3.7 software was used to evaluate nutrients.

At beginning (T0) and at the end of each treatment (T1, T2 and T3), all patients underwent body measurements, including weight, height, waist, abdomen, and hip circumferences.

At each checkpoint, participants underwent an US estimation of the body fat at xyphoid and umbilicus. Liver size and degree of liver steatosis was also measured. Fasting blood samples were collected at beginning (T0) and at the end of each treatment (T1, T2 and T3) to evaluate insulin resistance (IR, assessed by HOMA-IR), aminotransferases and gamma-glutamiltranspeptidase levels, lipid profile, oxidative stress (assessed by antioxidant enzyme activity, serum levels of MDA and carbonylated proteins) and inflammatory state (assessment of cytokine levels, typing of lymphocyte subpopulations, and metabolism).

Data collection was performed in a blind fashion: the statistician performing data analysis was blind to treatment. Access to data is available on the www.ELFID.unina.it website.

The primary outcome was a reduction of the liver steatosis estimated by US Scan, according to the following parameters: liver tissue echogenicity (compared with that the right kidney cortical), fat gain attenuation, and diaphragm blurring. The secondary outcomes were the reduction of IR, the improvement of serum lipid profile, the reduction of oxidative stress and the improvement in the inflammatory state.

### 2.2. Assessment of Anthropometric Status

Body weight was measured at the nearest 50 grams on the naked child. Abdominal circumference was measured at the level of the umbilicus, waist was measured at the approximate midpoint between the lower margin of the last palpable rib and the top of the iliac crest, while hip was estimated at the largest hip circumference around the widest portion of the buttocks.

### 2.3. Serum Lycopene Determination

Serum lycopene extraction and HPLC analysis were performed according to the slightly modified method by Tzeng et al. [19] described in detail in the Supplemental Information.

### 2.4. Oxidative Stress Determination.

Reduced and oxidized glutathione (GSH and GSSG, respectively) concentrations were measured in serum with the dithionitrobenzoic acid-GSSG reductase recycling assay. The GSH-to-GSSG ratio was used as oxidative stress marker. Carbonylated Proteins accumulation was measured according to Levine [20]. Malondialdehyde (MDA) levels were determined to assess lipid peroxidation [21]. Oxidized LDL were measured using commercially available ELISA kits (Human oxLDL 1 × 96 wellsCod. 10-1143-01 Mercodia, Sylveniusgatan, Uppsala, Sweden).

### 2.5. Ultrasound Monitoring

All subjects underwent echo-color Doppler ultrasound examination, using equipment iU22 Philips (Bothell, WA, USA) equipped with a Convex probe C5-2 MHz. Subcutaneous fat and visceral fat were measured at the sternum and at the umbilical level. The longitudinal diameter of the left lobe, the caudate lobe and the right lobe of the liver were measured. The time-gain compensation adjusted to maintain the tissue echogenicity as constant as possible regardless of the depth, and the power control was set at a constant level. Three parameters including parenchyma echogenicity, fat gain attenuation, and diaphragm blurring were assessed by visual evaluation comparing the echogenicity of the hepatic parenchyma with that of the cortical of the right kidney. In addition, a mini clip was acquired in a longitudinal scan on the right side of the line, including a scan on the liver and on the kidney.

Then, using the Q-Lab software, one ROI (region of interest) was drawn on the liver in an area with no vessels and no focal hyperechogenicity were visible, and another ROI was drawn on the renal cortex at the same depth as that of the liver. Hepato-renal index (HRI) was the ratio between liver and renal cortical echo amplitude, expressed in dB. The best cut off point for HRI was the value 2.2. Two experienced radiologists unaware of the patient’s clinical details and laboratory findings, performed US studies. The radiologists graded each US examination according to the presence and severity of liver steatosis by using the following criteria:

(1) Normal liver echo texture (absence of steatosis).
(2) Presence of hyperechogenic liver tissue (compared with the adjacent kidney cortex) with fine and tightly packed echo targets and of normal beam penetration with normal visualization of diaphragm and portal vein borders was considered as mild steatosis.
(3) Moderate and diffuse increase of echo intensity with decreased beam penetration (with slightly decreased visualization of diaphragm) associated with a decrease in visualization of silhouetting of the portal vein borders was considered as moderate steatosis.
(4) Marked increase in echoes intensity with no visualization of portal vein border, obscured diaphragm and posterior portion of the right lobe, and reduced visibility of kidney was considered as severe steatosis.

### 2.6. Measurement of Plasma Cytokine Levels

Interleukin (IL)-4, IL-6, IL-10 and TNF-α were measured by a Human Custom HS ProcartaPlex 4-plex kit (Invitrogen by Thermo Fisher Scientific, Waltham, MA, USA) according with manufacturer’s instructions; samples were analysed with a Luminex^®^ 200™. Human Adiponectin Platinum ELISA kit and Human Leptin Instant ELISA kit (both from Affimetrix–eBioscience, Santa Clara, CA, USA) were used for the quantitative detection of adiponectin and leptin, respectively.

### 2.7. Immunophenotypic and Flow Cytometry Analyses

Immunophenotypic analysis of peripheral blood was performed with an FC500 Flow Cytometer (Beckman Coulter, Brea, CA; USA) as previously described [22]. FACS analysis (FACSCanto II; BD Biosciences, Franklin Lakes, NJ, USA) of regulatory T cells in PBMCs was performed with the following: mAbs:CD4–allophycocyanin–H7 (RPA-T4) and CD25–PE–Cy7 (M-A251) (both from BD Pharmingen, Franklin Lakes, NJ, USA). Thereafter, cells were washed, fixed, and permeabilized (Human FoxP3 Buffer Set; BD Pharmingen) and were stained with FOXP3-PE (259D/C7) (BD Pharmingen). Analyses were performed with FACSDiva (BD) and FlowJo (BD) software.

### 2.8. T cell Cultures

PBMCs (1 × 10^5^ per well) were cultured in 96-well flat-bottom plates in 200 μL of RPMI 1640 medium supplemented with 5% autologous subject serum and were stimulated or not for a total of 60 h with anti-CD3 mAb (OKT3). After 48 h of stimulation, [^3^H]thymidine (0.5 μCi per well; Amersham-Pharmacia Biotech, PerkinElmer, Waltham, MA, USA) was added to the cell cultures, and cells were harvested 12 h later. Radioactivity was measured with a β cell plate scintillation counter (Wallac, Turku, Finland).

### 2.9. Bioenergetics and Metabolism of T Lymphocytes

Real-time measurements of the extracellular acidification rate (ECAR) and the oxygen consumption rate (OCR) were performed by an XF^e^96 Analyzer (Seahorse Bioscience, Billerica, MA, USA). PBMCs were cultured in medium or stimulated with anti-CD3 (OKT3) (4 × 10^5^ cells per well in 96-well culture plate) in 200 μL of RPMI 1640 medium supplemented with 5% autologous subject serum and incubated at 37 °C for 12 h. OCR was measured in XF media (non-buffered DMEM medium, containing 10 mM glucose, 2 mM L-glutamine, and 1 mM sodium pyruvate), under basal conditions and in response to 5 μM oligomycin, 1.5 μM of carbonylcyanide-4-(trifluoromethoxy)phenylhydrazone (FCCP) and 1 μM of antimycin and rotenone (all from Sigma Aldrich, St. Louis, MO, USA). Indices of mitochondrial respiratory function were calculated from OCR profile: basal OCR (before addition of oligomycin), ATP-linked OCR (calculated as the difference between basal OCR rate and oligomycin-induced OCR rate) and maximal OCR (calculated as the difference of FCCP rate and antimycin + rotenone rate). ECAR was measured in XF media in basal condition and in response to 10 mM glucose, 5 μM oligomycin and 100 mM of 2-DG (all from Sigma Aldrich). Indices of glycolytic pathway activation were calculated from ECAR profile: basal ECAR (after the addition of glucose), maximal ECAR (after the addition of oligomycin) and glycolytic capacity (calculated as the difference of oligomycin-induced ECAR and 2-DG-induced ECAR). Experiments with the Seahorse system were done with the following assay conditions: 3 min mixture; 3 min wait; and 3 min measurement. Metabolic parameters were then calculated. Data are expressed as mean and s.e.m.

### 2.10. Statistical Analysis

Data were inspected for normality and paired t-test (before/after) of each phase of the trial were performed when appropriate. A Bonferroni correction was applied to control for multiple comparison, within each domain. The mean percentage change of each variable between the values at 60 days (T1), 120 days (T2) and 180 days (T3) versus baseline (T0) values was estimated. Correlations among variables was estimated by the ‘*r*’ Pearson Correlation Coefficient. Distribution of US scores across the phase of the trial was compared by the chi square test.

Stepwise discriminant analysis was applied to the differences of all variables at time 60 days (end of the first trial) versus those at baseline, in order to sort out the most effective variables to distinguish between the two treatments. The Wilk’s Lambda (W) estimates the capacity of each variable to separate two groups, ranging from 1 = complete overlap to 0 = complete separation.

## 3. Results

Sixty-one obese children with liver steatosis were enrolled, 27 were randomly assigned to a restricted calorie regimen (RCR) alone (group 1), 34 to RCR with tomato juice supplementation (group 2). Two patients of group 1 and eight from group 2 withdrew before the trial period ended, 52 completed the study. The baseline characteristics of patients are shown in Table S1.

### 3.1. Diet

The average amount of daily calories (mean 1180) and all nutrients were not different between groups, according to the phase of the trial, with the exception of β-carotene, α-tocopherol, and insoluble fibers, which were higher during tomato supplementation (Table S2).

Serum lycopene was measured in a random sample of 9 children from Group 2 at T0, T1, and T2. As shown in Figure S2, its level significantly increased following 60 days (T1) of tomato juice supplementation compared to the baseline level (T0). At T2, after switching to solely RCR feeding, it was still high, although at a lesser extent.

### 3.2. Body Parameters

**Figure 1:**
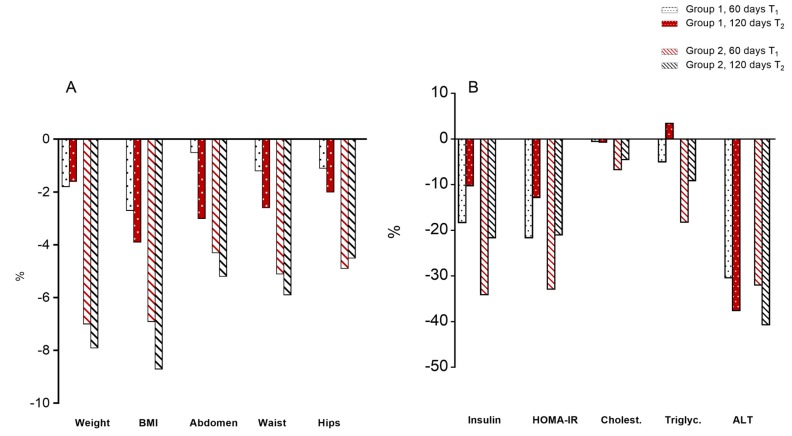
Percentage changes of anthropometric measures (**A**) and serum biochemical parameters (**B**) at 60 and 120 days compared to baseline (T0) (see study design). White = RCR; Red = RCR + tomato. Statistically significant differences are reported in Tables S3 and S4.

As shown in Figure 1A and in Table S3, body weight decreased significantly (*p* < 0.01 paired *t*-test) in both groups at T1, but more in the RCR + Tomato group: children on RCR + Tomato lost an average of 4 kg in the first 60 days (T1), while those on RCR lost an average of 1 kg. When children from group 2 (RCR + Tomato) switched to RCR (T2), they lost 610 grams on average, while group 1 (RCR), switched to RCR + Tomato (T2) did not change appreciably body weight after 60 days.

Apparently there were minor changes of BMI over the short time of the trial, but if we observe the percentage change over the baseline, it may be noted that children starting on RCR + Tomato decreased 7% of their BMI, compared to 2.7% of those on RCR-only at T1 (Figure 1A). Overall, the RCR + Tomato group kept a lower BMI across the trial.

All measures of abdominal fat (Abdomen, Waist, and Hip) decreased with caloric restriction, but the percentage decrease was about double for children starting with RCR + Tomato compared to the group on RCR only. Notably, also children from group 1 switched on RCR + Tomato (T2) decreased all the measures of the abdominal fat, even if at a minor rate compared to the group who started with RCR + Tomato (Figure 1A).

### 3.3. Serum Parameters

Serum parameters are shown in Figure 1B and Table S4. Glucose did not vary in both groups at all timepoints. Insulin levels showed a not significant decrease in group 1 compared to baseline (−18%), while group 2 showed a significant reduction in serum insulin at 60 days (T1) (−34%) which remained low for the subsequent time points (T2).

HOMA-IR, significantly decreased in group 1 (−21%), but even more in group 2 at T1 (−32%), then remained low for the subsequent time points (T2) in both groups.

Total cholesterol showed a decrease only in group 2 at T1 (−6.7%), followed by a slightly increase at T2. Notably, in group 2 LDL cholesterol was lower at T1 (−8.3%), while HDL increased in both groups at T1 and T2. Triglycerides decreased −18.2% in group with RCR + Tomato and −5% in group with RCR only. ALT and AST fell considerably (from −20 to −40%) in both groups (Table S4).

### 3.4. Oxidative State

**Figure 2:**
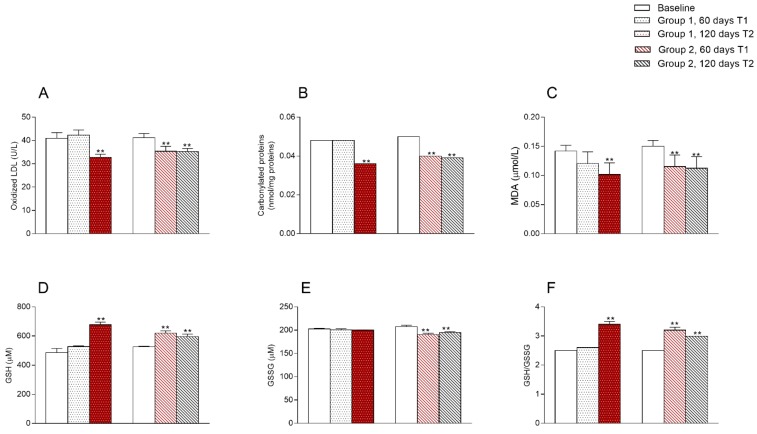
Changes in oxidative stress markers at 60 and 120 days compared to baseline. White = RCR, Red = RCR + tomato; Serum levels of (**A**) oxidized low-density lipoprotein (LDL); (**B**) carbonylated proteins (PC), (**C**) malondialdehyde (MDA); (**D**,**E**) reduced (GSH) and oxidized glutathione (GSSG) and (**F**) GSH to GSSG ratio. ** *p* < 0.01 paired Student’s-test comparing the effect of each intervention to the baseline. Oxidized LDL and carbonylated proteins were significantly reduced in both groups over tomato supplementation (Figure 2A,B). MDA decreased in both groups at T1 compared to T0 (Figure 2C) (−23% *p* = 0.000 for RCR + Tomato group, –15% *p* = 0.000 for RCR group), GSH increased and GSSG decreased in group 2 (RCR + Tomato), both at T1 and T2, compared to baseline values (Figure 2D,E). Consequently, GSH/GSSG ratio increased in group 2 at both time points (Figure 2F). In group 1 (RCR) GSH and GSH/GSSG ratio increased only at T2 (after RCR + Tomato) compared to baseline values, whereas GSSG did not change (Figure 2D–F).

Oxidative stress markers are reported in Figure 2.

### 3.5. Ultrasound Parameters

#### 3.5.1. Body Fat

**Figure 3:**
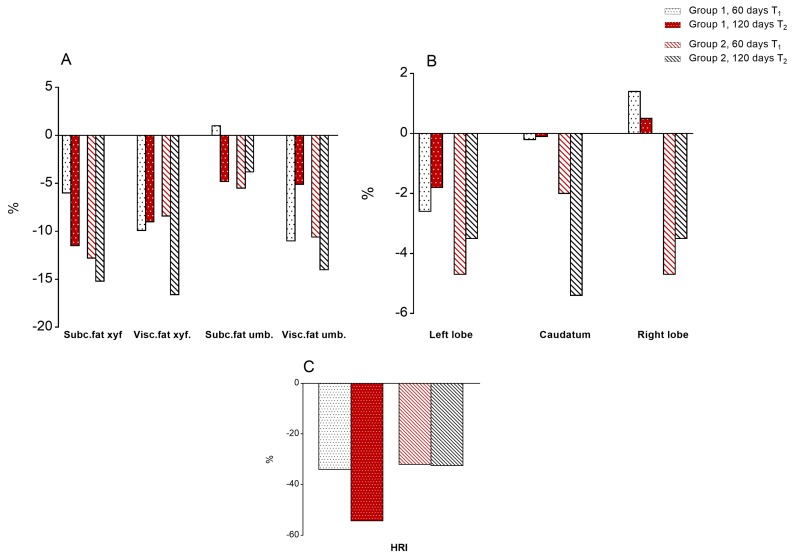
Percentage changes in ultrasound parameters at 60 and 120 days compared to baseline. Statistically significant differences are reported in Table S6. (**A**) Body fat; (**B**) Liver size; (**C**) Hepato-Renal Index, HRI. White = RCR, Red = RCR + T.

The loss of subcutaneous and visceral fat was always higher in children of group 2, up to the end of the trial (Figure 3 and Table S5). Indeed, at T2 the estimation of subcutaneous and visceral fat at xyfoid and umbilicus showed a marked decrease (about 16%) in group 2 (RCR + Tomato) and a lower decrease in group 1 (−10%).

#### 3.5.2. Liver Size

Despite the relatively short duration of the trial, the size of the liver, estimated at right, left and caudatum lobes, showed a greater decrease in group 2 than group 1 at T2 (Figure 3 and Table S5).

#### 3.5.3. Degrees of Steatosis

The ratio of reflectance of the liver tissue compared to the kidney cortex (Hepato-Renal Index, HRI) decreased, from −25 to −50%, in both groups in the first 60 days, and up to the end of trial (Figure 3 and Table S5).

When we compared the distribution of the degrees of steatosis (US scores) between T0 and T1 (60 days of treatment) we observed significant changes in both groups of children: in group 2, starting with RCR + Tomato, there were at baseline eight cases of severe steatosis, that were reduced to 3 at T1 and to only one at T2. 

**Figure 4:**
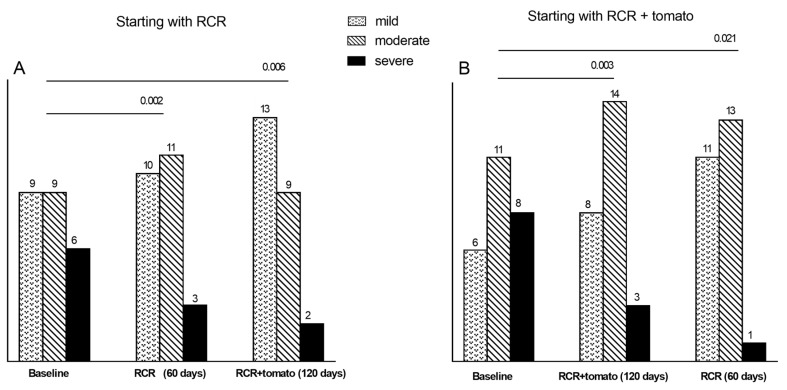
Stratification of patients by degrees of steatosis based on ultrasound findings at the baseline, 60 and 120 days. (**A**) group 1; (**B**) group 2.

Similarly, the six cases with severe steatosis at baseline in group 1, starting with RCR, decreased to three after 60 days (T1) and to only one at T2. In parallel, the cases with mild steatosis increased from six to 11 and from nine to 13 in group 2 and group 1, respectively (Figure 4).

### 3.6. Immunological and Inflammatory Profile

**Figure 5:**
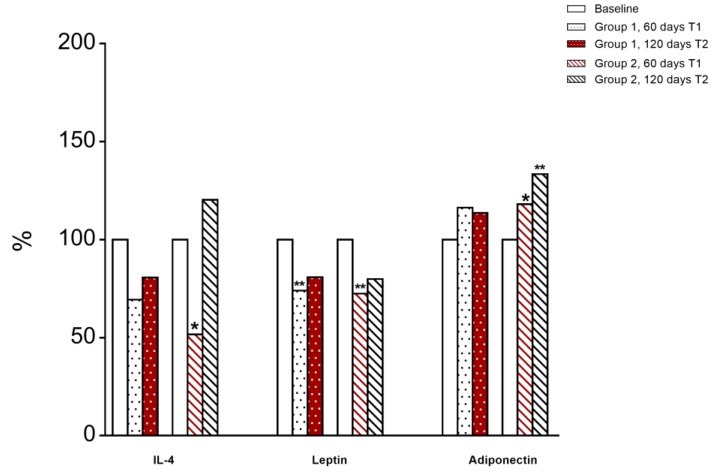
Percentage changes in serum IL-4 and adipokines levels at 60 and 120 days compared to baseline. White = RCR, Red = RCR+T. * *p* ≤ 0.05, ** *p* ≤ 0.01 Wilcoxon-test comparing the effect of each intervention to the baseline.

Levels of serum leptin, adiponectin, IL-4, IL-6, IL-10 and TNF-α were assessed. We observed a significant decrease of IL-4 at T1 in group 2 (RCR + Tomato) compared to T0, however, when children switched to RCR (T2) an increase in IL-4 serum level was shown (Figure 5).

At T1, leptin was significantly decreased in both groups of children compared to T0 (Figure 5).

Group 2 children (RCR + Tomato) displayed a significant increase in plasma adiponectin levels at T1 compared to T0; then after switching to RCR their serum level was further increased at T2 (Figure 5). No significant changes were observed in circulating levels of Il-6, IL-10, and TNF-α in both groups of children (data not shown).

We analysed the absolute number and the percentage of different immune cell subpopulations in peripheral blood of children at different timeline (Table S6). The analysis revealed a trend toward an increase of memory compartment cells (CD3^+^CD45RO^+^ and CD4^+^CD45RO^+^) in children from group 2 (RCR + Tomato). This increment was also observed in children of group 1 (RCR) when were supplemented with tomato juice.

We next evaluated the percentage of Foxp3^+^ regulatory T (Treg) cells in peripheral blood of the two groups of children at different time points. We did not observe a major change in the percentage of this cellular subset (Table S6).

To assess the effect of RCR alone and with tomato supplementation on the in vitro proliferation and metabolism of T cells, we purified peripheral blood mononuclear cells (PBMCs) from children of the two groups. The proliferation of PBMCs upon T cell receptor (TCR) anti-CD3 stimulation (OKT3) was inhibited in children of group 1 (RCR) at T1, this inhibition was not observed at T2 after switch to tomato (RCR + Tomato). Also in children of group 2 (RCR + Tomato), we observed a tendency to a reduction of PBMCs proliferation when they shifted to RCR at T2 (Table S6).

### 3.7. Mitochondrial Bioenergetics of T Lymphocytes

**Figure 6:**
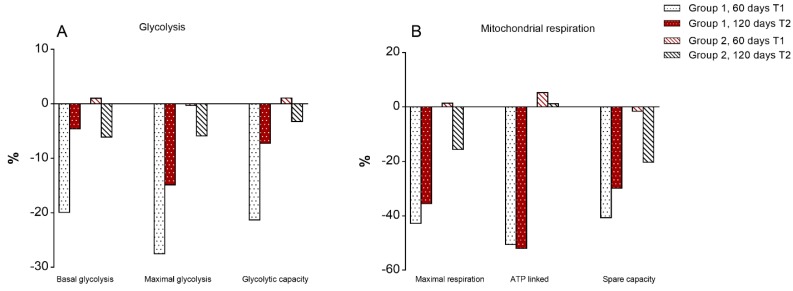
Percentage changes in metabolic parameters (**A**) Glycolysis; (**B**) Mitochondrial respiration) of PBMCs isolated from children at 60 and 120 days compared to baseline upon 12 h OKT3 stimulation. White = RCR, Red = RCR + Tomato. Statistically significant differences are reported in Figure S3.

We next analysed the extracellular acidification rate (ECAR) and the oxygen consumption rate (OCR), indicators of glycolysis and oxidative phosphorylation respectively, in PBMCs isolated from children of the two groups at T1 and T2 upon 12h OKT3 stimulation (Figure 6A,B, Figure S3).

In agreement with data on T cell proliferation, no change was observed in the cellular metabolism of children of group 2 (RCR + Tomato) at T1 and T2, while children of group 1 were characterized by a reduction of glycolytic metabolism, as shown by significant reduction of basal and maximal glycolysis and glycolytic capacity, together with a reduction of maximal respiration at T1. At the same time, there was a trend towards a reduction of glycolytic metabolism and mitochondrial respiration in children of group 2 when switched to RCR at T2 (Figure 6A,B, Figure S3).

### 3.8. Multivariate Analysis of Differences between T1 and T0

A multivariate discriminant analysis of the differences (D) between the end of the first trial (T1, 60 days) versus the baseline values in children starting with RCR + Tomato (group 2), versus those of RCR only (group 1), showed that the best discriminant variable is the decrease in visceral adiposity, as estimated by waist circumference (Wilk’s lambda = 0.668), followed by oxidative stress, estimated by the decrease in malondialdehydes (W = 0.496), then T cell metabolism was entered, estimated by oxygen consumption rate (W = 0.467), finally the decrease in body weight (W = 0.434) and the lowering of ALT levels (W = 0.400) were added (Table 1). 

This small set of variables, among the dozen examined, allows to correctly classify to each group 1 or 2, about 84% of children. However, the model was not developed to predict the well-known treatment, but to estimate the relative weight of the selected variables in differentiating the two treatments.

## 4. Discussion

Our results provide evidence that the dietary supplementation of tomato juice enriched with lycopene, eugenol, carvacrol, and thymol not only ameliorates glucose and lipid metabolism in obese children affected by NAFLD, but improving oxidative state and inflammatory markers, modulates the mitochondrial metabolism of T-lymphocytes contributing to a maintenance of a proper immune surveillance in children on RCR.

After 60 days of intervention (T1), children of group 1 on dietary restriction only (RCR), lost on average 1 kg, while those of group 2, on RCR supplemented with tomato juice, lost 4. BMI decreased about 2.7% in group 1 on RCR and about 7% in group 2 on RCR + Tomato. Waist circumference decreases by 2% in group 1 and by 5.1% in group 2. Previous studies showed that weight loss is the most effective strategy in the resolution or, at least in the short-term, improvement of NAFLD [23].

In our study HOMA-Index improved significantly with the RCR (−21%), but even more with tomato juice supplement (−33%). This effect is associated to a statistically significant decrease of leptin level in both groups and to an increase of adiponectin in group 2.

It is well known that obesity and in particular visceral fat, are linked to impaired glucose tolerance. A prominent role of visceral adipose tissue on the control of insulin resistance and inflammation has been showing obesity and metabolic syndrome [24]. Visceral adipose tissue secretes a number of adipokines, some of which are pro-inflammatory, such as leptin, which upregulates inflammatory cytokines (as TNF-α and IL-6) and others, such as adiponectin, which have an anti-inflammatory effect [25]. Abdominal obesity is related to adipokines imbalance: increased waist circumference is associated to decreased adiponectin and increased leptin serum levels [26]. Increased serum leptin levels correlate with severity of liver disease in NAFLD [27]. Leptin is thought to participate in both NAFLD/NASH progression, by contributing to the development of insulin resistance (IR) and steatosis and subsequently inducing fibrosis [10].

Dysregulated adipokine secretion causes macrophage infiltration into adipose tissue, leading to a low-grade chronic inflammation [28], which is associated with IR [29]. Adiponectin acts as an anti-inflammatory adipokine, as it exerts its effects by inhibiting NF-κB activation in endothelial cells and interfering with the function of macrophages. Decreased plasma concentration of adiponectin is associated with metabolic syndrome, whereas increased circulatory levels result in the improvement of insulin sensitivity. Indeed, serum adiponectin negatively correlates with percentage of body fat and rises with weight loss [30], nevertheless children supplemented with tomato juice, but not children on RCR, displayed an increase in plasma adiponectin levels. Moreover, tomato supplementation was shown to enhance adiponectin circulatory levels in young women independently of body fat reduction, suggesting a direct effect of tomato supplementation on adipokine expression [31]. A significant increment of adiponectin mRNA expression and plasma concentration was observed also in obese rats on HFD supplemented with lycopene, compared to rats solely fed with HFD, although both body weight and adiposity were not different between groups, strengthening a direct effect of tomato supplement on adiponectin transcription. In fact, mRNA levels of *SIRT1* and *FoxO1*, both involved in transcriptional regulation of adiponectin, were upregulated in adipose tissue of the rats on HFD supplemented with tomato juice, suggesting that lycopene-induced upregulation of *SIRT1* and *FoxO1* might mediate adiponectin gene expression [32].

Metabolic effects of tomato supplementation are not limited to the control of glucose homeostasis, but also extend to lipid metabolism, as already reported [33]. In our study, total cholesterol and triglycerides were lower in RCR associated to tomato supplement than RCR alone (−6.7% vs. −0.5% and −18.3% vs. −5% respectively). In the RCR + Tomato group the reduction of triglycerides and LDL significantly correlates with the decrease of ALT (*r* = 0.658 *p* = 0.000 and *r* = 0.523 *p* = 0.012, respectively) and is inversely related to HRI values (*r* = −0.427 *p* < 0.05), suggesting that the improvement of steatosis is related to that of lipid metabolism. The thickness of the fat deposits, estimated by US, decreased by 5–10% in children on RCR, while it decreased by 1020% when tomato juice was administered; similarly, the size of the liver decreased in children on RCR, and even more in RCR supplemented with tomato juice.

Development of obesity in mice was correlated with a change in cytokine profile with decreased IL-2 and increased IFN-γ and IL-4 [34]. In children on RCR supplemented with tomato juice, we observed a significant decrease of IL-4 compared to the children on diet only. Consistently, several studies have reported the anti-inflammatory effects of RCR and tomato phytonutrients, such as lycopene and β-carotene [35,36]. The ability of lycopene to reduce pro-inflammatory cytokine and chemokine expression, by modulating NF-κB signalling pathway, was displayed in ex vivo cultures of adipose tissue explants from HFD-fed mice and in human adipocyte cultures [37].

Recent study demonstrated that in obesity conditions the alterations in redox homeostasis and oxidative stress are present from an early age, as indicated by an increase in MDA content and GSH/GSSG ratio [38]. Malondialdehyde (MDA), an important biomarker of oxidative stress, is generated by the peroxidation of lipids containing polyunsaturated fatty acids and is cytotoxic due to its ability to induce macromolecular crosslinking polymerization [39]. GSH-GGSG ratio is an important biomarker of the redox state and consequently of the oxidative stress [40]. Here, we showed that MDA, oxidized LDL and carbonylated proteins were lower in serum of children on RCR + Tomato than in those on RCR alone, while GSH was higher in the RCR + Tomato children when compared to RCR alone. In the tomato-supplemented children, the improvement of oxidative stress biomarkers lasted up to two months after the switch to RCR. Most variables indeed showed a carry-over effect across the change of diet. It was demonstrated that GSH plays an important role in maintenance of numerous immune functions including lymphocyte proliferation and NK cell activity [41]. Several clinical trials on RCR with tomato products showed an increase in antioxidant defences, in healthy humans [42] as well as in obese or diabetic patients [43].

Changes in metabolic and nutritional status of the whole organism were shown to enhance or suppress specific T cell functions [8]. Several data highlighted the key role of metabolic pathways in the regulation of immune responses [9]. Upon antigen encounter and activation, naïve T cells undergo rapid proliferation and differentiation into effector T cells able to identify and eliminate pathogens. These changes in growth, proliferation and functions need to be sustained by a metabolic activation characterized by increased nutrient uptake and switch to a glycolytic metabolism. We observed that T cells from children on RCR had a reduction in glycolytic metabolism and proliferation. These data suggest that the reduction of nutrient uptake for 60 days is associated with impaired engagement of T cells glycolysis and proliferation related to a reduced capacity of the immune system to respond against possible foreign antigens [44,45].

Our results are in agreement with data suggesting that, although dietary restriction improves health and delays tissue aging, it impairs hematopoietic stem cells (HSC) differentiation into lymphoid lineages, inhibits the proliferation of lymphoid progenitors [46] and increases mortality of mice in response to different pathogens when compared to *ad libitum* fed counterparts [47].

Interestingly, daily supplementation with lycopene-enriched tomato juice resulted in the maintenance over time of the physiological engagement of glycolytic metabolism in T cells after activation, despite the caloric restriction. The glycolytic metabolic activation of T cells has to be associated with the maintenance of a proper immune surveillance in children on calories restricted diet. Taken together, these results provide promising rationale for the potential use of tomato juice supplementation to enhance antioxidant capacity and immune functions in obese children affected by NAFLD.

In conclusion, we confirmed that the restriction of calories is an optimal therapy to slow down the progression of NAFLD in obese children, but the addition of a tomato supplement, typical choice of the Mediterranean Diet, significantly strengthens the action of the caloric restriction and protects the individual from the constraints of the energy restriction in a growing organism. The addition of tomato juice or any other form of tomato supplementation to a calorie restricted diet in NAFLD children is generalizable.

## 5. Study Strengths and Limitations

The strength of this study is the crossover design and the extensive evaluation of outcomes (body weight, fat, liver, biochemistry, lipids, oxidative stress, cytokines, t-cell population and metabolism); limitation of the study is the lack of liver biopsy and no long term data. A carry over effect was observed over the change of the dietary regimen: the starting treatment in the first phase (RCR or RCR + Tomato) imprinted the results of the subsequent phases of the trial. This intervention is dedicated to obese NASH children, many of the observed improvements in the metabolic status of NAFLD are limited to the administration of the caloric restricted diet with/without tomato supplement, but it is clear that the addition of tomato could well be considered a protective and preventive support to obese child.

## Figures and Tables

**Table 1:** Multivariate discriminant analysis showing the best variables able to discriminate RCR from RCR+Tomato groups at the end of the first phase of the trial.

Step	Differences between Values at 60 Days Versus Baseline	Wilks Lambda *	Exact F *P*	
1	D-WAIST	0.668	10.415	0.004
2	D-MDA	0.496	10.146	0.001
3	D-Mitochondrial OCR	0.467	7.238	0.002
4	D-WEIGHT	0.434	5.873	0.003
5	D-ALT	0.400	5.099	0.005

* cumulative capacity to discriminate of the model.

## Supplementary Materials

The following are available online at https://www.mdpi.com/2077-0383/9/1/141/s1, Figure S1: CONSORT Flow Diagram, Figure S2: Serum lycopene levels determined in group 2 (RCR + Tomato), Figure S3: Metabolic parameters of PBMCs isolated from children at 60 and 120 days compared to baseline (T0) upon 12 h OKT3 stimulation, Table S1: Demographic characteristics of patients, Table S2: Daily energy intake and RCR composition, Table S3: Body parameters at baseline and after 60, 120 and 180 days, Table S4: Serum parameters at baseline and after 60, 120 and 180 days, Table S5: Ultrasound parameters at baseline and after 60, 120 and 180 days, Table S6: Absolute number and percentage of different immune cell subpopulations in peripheral blood of children at baseline and after 60 and 120 days.
