# Supplementary material for: Randomised Clinical Trial: Calorie Restriction Regimen with Tomato Juice Supplementation Ameliorates Oxidative Stress and Preserves a Proper Immune Surveillance Modulating Mitochondrial Bioenergetics of T-Lymphocytes in Obese Children Affected by Non-Alcoholic Fatty Liver Disease (NAFLD)
Source: J Clin Med. 2020 Jan 4;9(1):141. doi: 10.3390/jcm9010141 (PMC7019306; doi:10.3390/jcm9010141)
Supplement: Supplementary file 1 [file jcm-09-00141-s001.pdf]

Supporting information

Figure S1. CONSORT flow diagram.

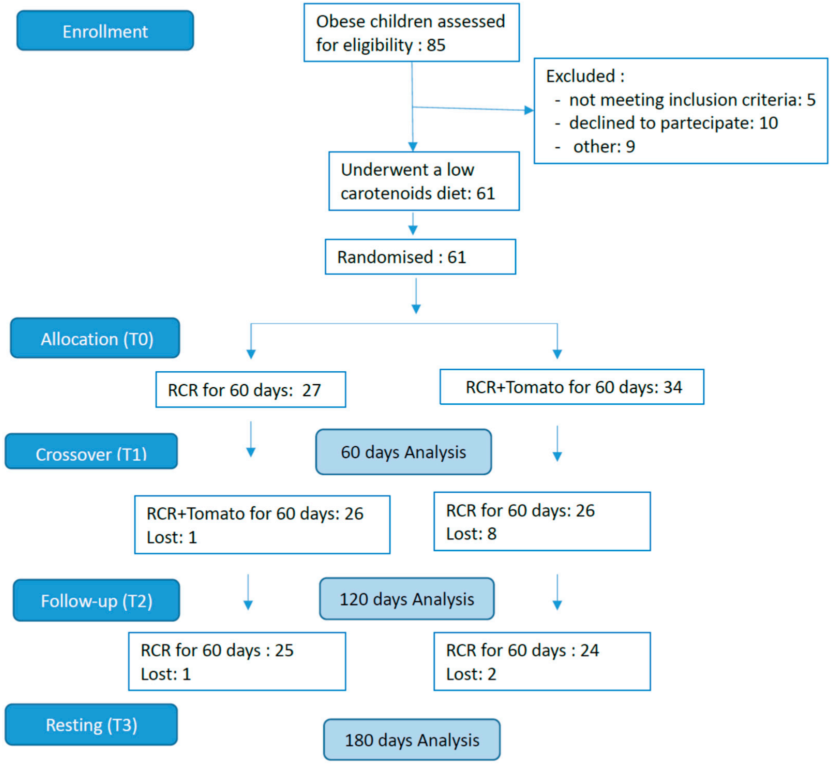

Serum lycopene levels determination

200 μL of serum were mixed with 1 mL of distilled water and 1 mL of ethanol solution containing 0.01% ascorbic acid for the precipitation of protein and protection of lycopene. Two mL of hexane was added for the extraction of lycopene, and the mixture was mixed and centrifuged at 3000 rpm for 7 min, after which the hexane layer was collected and evaporated to dryness under nitrogen (for three times). The residue was dissolved in 200 μL of dichloromethane /methanol (45:55, v/v) and 10 μL were injected into an HPLC system (Agilent 1260 Infinity) equipped with autosampler. Lycopene separation was performed using an analytical column (Agilent Eclipse Plus C18 3.5 μm, 2.1 x 150 mm) maintained at room temperature without guard column. The mobile phase was composed of dichloromethane (solvent A) and methanol (solvent B) and the elution was achieved using the following conditions: (1) isocratic elution with 5% A from the injection time until 11 min, (2) a gradient from 5% A to 50% A for 1 min, (3) 50% A for 2 min, (iv) a gradient from 50% to 5% A for 1 min, (v) 5% A over 8 min (total run time 23 min). The detection wavelength was set at 476 nm, and the flow rate was 0.4 mL/min. Lycopene was quantified using a standard calibration curve ranging from 0 to 1000 ng/mL, as previously described [16]. Data are reported as average and standard deviation (SD).

Anthropometric measures, serum biochemical levels, ultrasound parameters and HRI at T3 phase

In table S3 and S4 we reported the % mean change of anthropometric measures and serum biochemical parameters at 60, 120 and 180 days compared to baseline (T0). These results highlight that during the resting phase T3 (further 60 days on RCR for both groups), children from group 1 returned to the baseline body weight, while group 2 weighed 5 kg less that the baseline value. Moreover, at the end of the resting phase BMI was decreased -8.9% in children started with RCR+Tomato and -1.7% in those starting with RCR only (Table S3).

Insulin levels and HOMA-IR remained low for all time points in both groups (Table S4). Notably at T3, in group 2 total cholesterol returned to baseline values, while HDL increased in both groups at T1, T2 and T3 (Table S4).

The loss of subcutaneous and visceral fat was always higher in children of group 2, up to resting phase (T3) (Table S5). Indeed, at T3 the estimation of subcutaneous and visceral fat at xyfoid and umbilicus showed a marked decrease (about -20%) in group 2 (RCR+Tomato) and a lower decrease in group 1 (-10%). The ratio of reflectance of the liver tissue compared to the kidney cortex (Hepato-Renal Index, HRI) decreased in both groups up to the end of trial (T3) (Table S5).

Figure S2. Serum Lycopene levels determined in group 2 (RCR+Tomato).

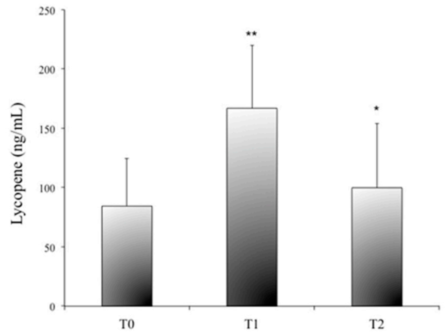

Mean serum lycopene levels (ng/mL) determined at baseline (T0), 60 days (T1) and 120 days (T2) in a random sample of 9 children. ** T1 versus T0, $p = 0.005$; * T2 versus T1, $p = 0.030$ (ANOVA with Bonferroni post hoc test). Error bars correspond to standard deviations.

Table S1. Demographic characteristics of patients.

Variable	Group 1 (26)	Group 2 (35)
Age	10.8 ± 1.9	10.4 ± 2.1
Sex m	10	23
f	16	12
BMI centiles	94.8 ± 2.9	95.3 ± 2.7
Height centiles	77.9 ± 24.9	68.9 ± 26.1
Weight centiles	94.9 ±3.3	93.9 ± 6.6

Commented [M1]: If the number has a decimal point in the middle,
please change the comma to a decimal point in this image.
For example, 10,8 should be changed to10.8. Please revise the table.

Table S2. Daily energy intake and RCR composition. Group 1: RCR; Group 2: RCR+Tomato. Student's T-test.

	Group	Mean	SD	p
**Energy (Kcal)**	1	1175.3	186.9	
	2	1180.7	193.8	0.919
**Proteins (g)**	1	53.5	11.4	
	2	53.0	11.7	0.864
**Total Lipids (g)**	1	38.1	7.0	
	2	40.7	8.4	0.241
**Simple sugars (g)**	1	164.6	35.1	
	2	160.2	34.6	0.646
**Olygosaccharides (g)**	1	49.7	16.6	
	2	46.5	19.6	0.527
**Insoluble Fibers (g)**	1	4.2	2.0	
	2	5.8	3.1	0.036
**Cholesterol (mg)**	1	142.9	78.6	
	2	168.7	77.1	0.236
**Saturated FA (mg)**	1	9.8	2.5	
	2	10.4	2.7	0.424
**Polyunsaturated FA (mg)**	1	4.6	0.9	
	2	4.8	1.2	0.550
**Monounsatured FA (mg)**	1	20.3	5.0	
	2	21.7	4.8	0.293
**Polyphenols (mg)**	1	620.2	299.9	
	2	515.5	235.1	0.160
**Beta-Carotene (mg)**	1	902.0	785.3	
	2	1878.1	1588.0	0.008
**Alpha-Tocopherol (mg)**	1	0.59	0.33	
	2	0.88	0.65	0.058

Commented [M2]: If the number has a decimal point in the middle,
please change the comma to a decimal point in this image.
Please revise the table.

Table S3. Body parameters (mean ±SE and % change from baseline) at baseline and after 60, 120 and 180 days.

Variable	Group	Baseline	Treatment	Day 60	%mean change	Treatment	Day 120	% mean change	Treatment	Day 180 §	% mean change
Weight (Kg)	1	63.1 ± 3.0	RCR	61.9 ± 2.9**	-1.8	RCR+TOMATO	62.0 ± 3.0*	-1.6	RCR	64.4 ± 3.2	2.0
	2	60.1 ± 2.8	RCR+TOMATO	55.9 ± 2.7**	-7.0	RCR	55.3 ± 3.4**	-7.9	RCR	55.4 ± 3.6	-7.8
BMI	1	26.7 ± 0.7	RCR	26.0 ± 0.7**	-2.7	RCR+TOMATO	25.7 ± 0.8**	-3.9	RCR	26.3 ± 0.7	-1.7
	2	26.6 ± 0.7	RCR+TOMATO	24.7 ± 0.6**	-6.9	RCR	24.2 ± 0.7**	-8.7	RCR	24.2± 0.9	-8.9
Waist (cm)	1	83.4 ± 1.7	RCR	82.5 ± 1.6*	-1.2	RCR+TOMATO	81.3 ± 1.7**	-2.6	RCR	83.5 ± 1.6	0.0
	2	83.5 ± 1.6	RCR+TOMATO	79.2 ± 1.7**	-5.1	RCR	78.5 ± 2.3**	-5.9	RCR	78.4 ± 2.4*	-6.14
Hip (cm)	1	94.1 ± 2.1	RCR	93.1 ± 2.2	-1.1	RCR+TOMATO	92.2 ± 2.0*	-2.0	RCR	96.2 ± 2.2	2.2
	2	93.2 ± 2.1	RCR+TOMATO	88.7 ± 2.0**	-4.9	RCR	89.0 ± 2.4**	-4.5	RCR	89.5 ± 2.4	-4.0
Abdomen (cm)	1	93.3 ± 2.1	RCR	92.9 ± 2.3	-0.5	RCR+TOMATO	90.5 ± 2.2**	-3.0	RCR	93.0 ± 2.1	-0.3
	2	90.8 ± 1.9	RCR+TOMATO	86.9 ± 1.8	-4.3	RCR	86.1 ± 2.5	-5.2	RCR	85.6 ± 2.5	-4.8
% Body fat	1	21.7 ± 2.1	RCR	20.7 ± 2.0**	-4.6	RCR+TOMATO	19.4 ± 2.0**	-10.6	RCR	20.1 ± 1.8	-7.3
	2	24.4 ± 1.6	RCR+TOMATO	21.5 ± 1.6**	-11.9	RCR	19.5 ± 2.3**	-20.0	RCR	22.1 ± 2.6	-9.4

*: p≤0.05 **p≤0.01 paired Student's t-test comparing the effect of each intervention to the baseline

Commented [M3]: If the number has a decimal point in the middle,
please change the comma to a decimal point in this image.
Please revise the table.

Table S4. Serum parameters (mean ±SE and % change from baseline) at baseline and after 60, 120 and 180 days.

Variable	Group	Baseline	Treatment	Day 60	%mean change	Treatment	Day 120	%mean change	Treatment	Day 180	%mean change
Glucose (mg/dl)	1	77.1 ± 1.2	RCR	74.9 ± 1.7	-2.8	RCR+TOMATO	73.2 ± 1.4	-5.1	RCR	75.0 ± 1.8	-2.7
	2	75.7 ± 1.3	RCR+TOMATO	73.9 ± 1.4	-2.5	RCR	74.0 ± 1.4	-2.3	RCR	75.2 ± 1.5	-0.7
Insulin (mU/L)	1	21.0 ± 2.2	RCR	17.1 ± 1.4	-18.3	RCR+TOMATO	18.9 ± 1.8	-10.2	RCR	17.2 ± 2.2	-18.1
	2	21.3 ± 2.8	RCR+TOMATO	14.0 ± 1.9 **	-34.1	RCR	16.7 ± 2.9	-21.6	RCR	14.8 ± 2.8	-30.4
HOMA-IR	1	4.0 ± 0.4	RCR	3.2 ± 6.3*	-21.6	RCR+TOMATO	3.5 ± 0.3	-12.8	RCR	3.2 ± 0.4	-20.6
	2	3.9 ± 6.5	RCR+TOMATO	2.6 ± 6.4**	-32.9	RCR	3.1 ± 0.6	-21.0	RCR	2.8 ± 0.2	-29.1
Total cholesterol (mg/dl)	1	148.9 ± 5.8	RCR	148.2 ± 5.4	-0.5	RCR+TOMATO	147.8 ± 5.8	-0.7	RCR	154.0 ± 5.5	3.4
	2	152.9 ± 4.8	RCR+TOMATO	142.7 ± 5.5*	-6.7**	RCR	146.0 ± 7.0	-4.5	RCR	152.3 ± 8.9	-0.4
LDL cholesterol (mg/dl)	1	97.2 ± 6.3	RCR	94.7 ± 5.7	-2.5	RCR+TOMATO	90.6 ±5.8	-6.8	RCR	98.0 ±5.2	3.8
	2	97.3 ± 4.5	RCR+TOMATO	89.2 ± 4.9**	-8.3	RCR	85.9 ± 5.7**	-7.2	RCR	93.9 ± 7.2	-1.6
HDL cholesterol (mg/dl)	1	41.2 ± 1.5	RCR	41.3 ± 1.6	0.3	RCR+TOMATO	43.2 ± 1.7	4.8	RCR	45.4 ± 1.8	10.1
	2	40.7 ± 1.2	RCR+TOMATO	41.0 ± 1.9	0.8	RCR	42.3 ± 1.7	3.9	RCR	44.3 ± 2.5**	8.8
	1	*104.2 ±10.9*	RCR	98.9 ± 10.2	-5.0	RCR+TOMATO	107.8 ±14.7	3.4	RCR	96.8 ± 10.1	-7.5

Commented [M4]: If the number has a decimal point in the middle,
please change the comma to a decimal point in this image.
Please revise the table.

Triglyceride (mg/dl)	2	*98.4 ± 7.7*	RCR+TOMATO	80.5 ± 7.6**	-18.2	RCR	89.5 ± 17.4	-9.1	RCR	100.2 ± 19.2	1.8
ALT (UI/L)	1	36.1 ± 7.4	RCR	25.1 ± 2.67	-30.4	RCR+TOMATO	22.5 ± 2.2	-37.6	RCR	25.2 ± 2.2	-31.5
	2	71.3 ± 10.9	RCR+TOMATO	48.5 ± 8.1**	-32.0	RCR	42.3 ± 8.8*	-40.7	RCR	54.5 ± 18.6	-23.5
AST (UI/L)	1	30.5 ± 4.0	RCR	22.8 ± 1.2	-25.3	RCR+TOMATO	21.9 ± 1.2	-27.9	RCR	23.8 ± 1.3	-21.6
	2	44.3 ± 5.1	RCR+TOMATO	34.4 ± 3.9*	-22.3	RCR	31.0 ± 3.7	-30.0	RCR	35.9 ± 5.9	-18.2

*: p≤0.05 **p≤0.01 paired Student’s t-test comparing the effect of each intervention to the baseline

Table S5. Ultrasound parameters (mean ±SE and % change from baseline) at baseline and after 60, 120 and 180 days.

Variable	Group	Baseline	Treatment	Day 60	% mean change	Treatment	Day 120	% mean change	Treatment	Day 180	% mean change
Subcutaneous fat at xyfoid level	**1**	15.4 ± 0.8	RCR	14.5 ± 0.7	-6.0	RCR+TOMATO	13.6 ± 0.7	-11.5	RCR	13.8 ± 0.6*	-10.2
	**2**	13.6 ± 0.7	RCR+TOMATO	11.9 ± 0.7*	-12.8	RCR	11.6 ± 0.8	-15.2	RCR	11.1 ± 1.1	-18.7
Visceral fat at xyfoid level	**1**	13.0 ± 1.1	RCR	11.7 ± 0.9	-9.9	RCR+TOMATO	11.8 ± 0.7*	-9.0	RCR	12.8 ± 1.1	-1.4
	**2**	11.6 ± 1.1	RCR+TOMATO	10.6 ± 0.9	-8.4	RCR	9.7 ± 0.7	-16.6	RCR	9.3 ± 1.1	-19.5
Subcutaneous fat at umbilical level	**1**	20.7 ± 1.6	RCR	20.9 ± 1.5	1.0	RCR+TOMATO	19.7 ± 1.2	-4.8	RCR	21.2 ± 1.2	2.7
	**2**	18.7 ± 0.9	RCR+TOMATO	17.7 ±1.1	-5.5	RCR	17.0 ± 1.2	-3.8	RCR	16.6 ± 1.1	-2.3
Visceral fat at umbilical level	**1**	35.7 ± 2.1	RCR	31.8 ± 2.0	-11.0	RCR+TOMATO	33.8 ± 1.7	-5.1	RCR	33.1 ± 1.3	-7.1
	**2**	36.9 ± 2.1	RCR+TOMATO	33.0 ± 2.0	-10.6	RCR	31.8 ± 1.9	-14.0	RCR	33.7 ± 2.6	-8.7
Liver right lobe (mm)	**1**	146.0 ± 4.1	RCR	147. ± 3.3	1.4	RCR+TOMATO	146.5 ± 3.0	0.5	RCR	147.8 ± 3.1	1.4
	**2**	151.6 ± 3.7	RCR+TOMATO	144.4 ± 3.9	-4.7	RCR	143.9 ± 3.0	-3.5	RCR	149.9 ± 2.9	-1.7
Liver left lobe (mm)	**1**	100.9 ± 3.5	RCR	98.3 ± 2.7	-2.6	RCR+TOMATO	99.1 ± 3.0	-1.8	RCR	97.4 ± 3.1	-3.5
	**2**	102.0 ± 3.0	RCR+TOMATO	97.3 ± 2.6	-4.7	RCR	98.4 ± 3.0	-3.5	RCR	100.3 ± 3.5	-1.7
Liver caudatum (mm)	**1**	*44.8* ± 1.8	RCR	44.8 ± 1.3	-0.2	RCR+TOMATO	44.8 ± 1.5	-0.1	RCR	42.7 ± 1.5	-4.8
	**2**	*44.8* ± 1.6	RCR+TOMATO	43.9 ± 1.6	-2.0	RCR	42.4 ± 1.5	-5.4	RCR	45.4 ± 2.0	1.4
HRI	**1**	3.2 ± 0.2	RCR	2.15 ± 0.2*	-34	RCR+TOMATO	1.48 ± 0.1	-54.4	RCR	2.1 ± 0.2	-35.8

Commented [M5]: If the number has a decimal point in the middle,
please change the comma to a decimal point in this image.
Please revise the table.

	**2**	*2.6* ± 0.4	RCR+TOMATO	1.76± 0.4	-32	RCR	1.75	-32.5	RCR	1.92 ± 0.2	-25.8

*: *p*≤0.05 **$p \leq 0.01$ paired Student's t-test comparing the effect of each intervention to the baseline

Table S6. Absolute number and percentage of different immune cell subpopulations in peripheral blood of children at baseline and after 60 and 120 days.

Variable	Group	Baseline	Treatment	Day 60	Treatment	Day 120
Leucocytes cells	**1**	5925 ± 359	RCR	5608 ± 289	RCR+Tomato	6352 ± 370
	**2**	6075 ± 354	RCR+Tomato	5699 ± 273	RCR	5932 ± 410
Lymphocytes cells	**1**	2394 ± 123	RCR	2362 ± 128	RCR+Tomato	2520 ± 143
	**2**	2855 ± 161	RCR+Tomato	2716 ± 132	RCR	2700 ± 217
$CD3^{+}$ cells	**1**	1608 ± 90	RCR	1632 ± 99	RCR+Tomato	1779 ± 118
	**2**	2036 ± 120	RCR+Tomato	1971 ± 98	RCR	1955 ± 164
$CD4^{+}$ cells	**1**	847 ± 49	RCR	861 ± 55	RCR+Tomato	954 ± 62
	**2**	1052 ± 59	RCR+Tomato	1018 ± 45	RCR	1009 ± 78
$CD8^{+}$ cells	**1**	578 ± 39	RCR	584 ± 50	RCR+Tomato	636 ± 51
	**2**	743 ± 64	RCR+Tomato	724 ± 57	RCR	724 ± 68
$CD3^{+}CD45RA^{+}$ cells	**1**	952 ± 70	RCR	952 ± 70	RCR+Tomato	1044 ± 80
	**2**	1300 ± 96	RCR+Tomato	1162 ± 75	RCR	1182 ± 110
$CD3^{+}CD45RO^{+}$ cells	**1**	654 ± 47	RCR	677 ± 52	RCR+Tomato	736 ± 62
	**2**	736 ± 42	RCR+Tomato	809 ± 51	RCR	785 ± 69
% $CD3^{+}CD45RA^{+}$ cells	**1**	39 ± 2	RCR	40 ± 2	RCR+Tomato	41 ± 2
	**2**	44 ± 1	RCR+Tomato	43 ± 2	RCR	43 ± 1
% $CD3^{+}CD45RO^{+}$ cells	**1**	28 ± 1	RCR	29 ± 2	RCR+Tomato	29 ± 2
	**2**	27 ± 1	RCR+Tomato	30 ± 1	RCR	29 ± 1
$CD4^{+}CD45RA^{+}$ cells	**1**	454 ± 41	RCR	479 ± 40	RCR+Tomato	510 ± 40
	**2**	652 ± 48	RCR+Tomato	593 ± 40	RCR	619 ± 64
$CD4^{+}CD45RO^{+}$ cells	**1**	394 ± 22	RCR	383 ± 32	RCR+Tomato	444 ± 41
	**2**	400 ± 22	RCR+Tomato	425 ± 24	RCR	389 ± 23
% $CD4^{+}CD45RA^{+}$ cells	**1**	19 ± 1	RCR	20 ± 1	RCR+Tomato	20 ± 1
	**2**	23 ± 1	RCR+Tomato	22 ± 1	RCR	22.48 ± 1
% $CD4^{+}CD45RO^{+}$ cells	**1**	17 ± 1	RCR	16 ± 1	RCR+Tomato	17.5 ± 1
	**2**	15 ± 1	RCR+Tomato	16 ± 1	RCR	15.32 ± 1
% $CD4^{+}Foxp3^{+}$ cells	**1**	4 ± 0	RCR	4.8 ± 0	RCR+Tomato	4.29 ± 0.4

Commented [M6]: If the number has a decimal point in the middle,
please change the comma to a decimal point in this image.
Please revise the table.

	**2**	5 ± 0	RCR+Tomato	4.9 ± 0	RCR	5.38 ± 0.2
T cell anti-CD3 proliferation	**1**	64196 ± 6264	RCR	56966 ± 6878	RCR+Tomato	66448 ± 8227
	**2**	60149 ± 6343	RCR+Tomato	62567 ± 5162	RCR	48378 ± 4370

Figure S3 **Metabolic parameters of PBMCs isolated from children at 60 and 120 days compared to baseline (T0) upon 12h OKT3 stimulation. White = RCR, Red = RCR+T; *p<0.05, **p<0.01 paired Student's-test comparing the effect of each intervention to the baseline.**

Commented [M7]: Please explain the meaning of "*" in the Supplementary Fig. 3.

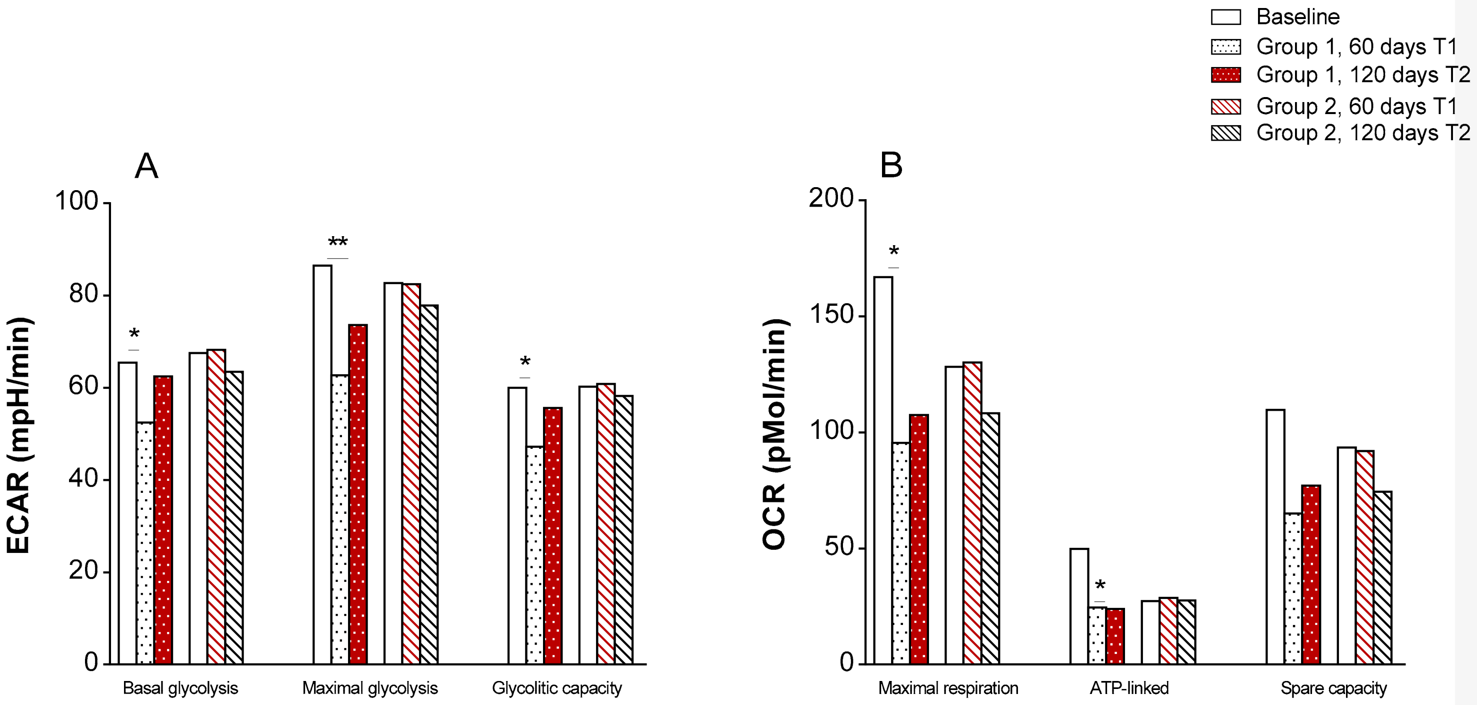

A. Glycolytic T cell metabolism after 60 and 120 days of treatment. Lower basal, maximal and glycolytic capacity from baseline was observed in children on RCR-only, whereas any change was observed in in children on RCR+Tomato. **B.** Together with a reduction of glycolytic

capacity, an impairment of oxidative metabolism was observed in children on RCR-only. Also children who started with RCR+Tomato, decreased their ECAR and OCR when switched to RCR-only.
